# Balancing the books

**Published:** 2013

**Authors:** Allen Foster

**Affiliations:** Co-director: International Centre for Eye Health, London School of Hygiene and Tropical Medicine, London, UK.

**Figure:**
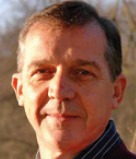
Allen Foster

Two critical questions face managers of eye care institutions:

- How can I reduce costs?
- How can I generate income?

## Costs/expenditure

The costs of any eye care service can be divided into:

- Development costs – these are one-time (or infrequent) costs
- Service provision (running) costs – these are ongoing (weekly, monthly, or annual) costs.

### Development costs

For the purpose of this article we will assume that development costs (equipment, instruments, vehicles, and training staff) will be financed by one-time investments from the government, non-governmental organisations (NGOs), local philanthropists or hospital savings. These development investments are very important; however, they are occasional and once made they are no longer critical to the ongoing financial sustainability of the eye care service.

### Service provision (running) costs

These ongoing costs include salaries, consumables, utilities (water and electricity), rent, maintenance, and depreciation costs.

**Figure:**
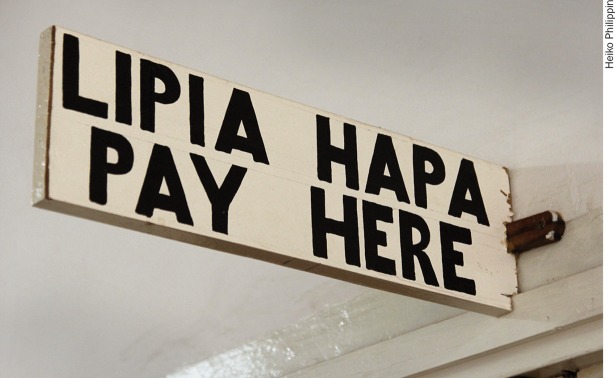
Patients contribute to the financial sustainability of eye services.

## How to reduce costs

One can reduce salary costs by only employing essential staff. Each employee should have a clear job description for which they are well trained. Annual performance reviews and objective setting, with non-monetary incentives for good performance, can create a positive work culture.

Increasing productivity does not reduce the salary bill. But, where patients pay for services, seeing more outpatients, dispensing more spectacles and performing more eye operations (in the same time and with the same staff) can improve the financial situation and the sustainability of the service.

One can reduce the cost of consumables by:

- Only purchasing essential consumables
- Purchasing in bulk
- Using generic drugs and other consumables, thereby avoiding expensive ‘brands’ or designer-labelled consumables
- Ensuring that the eye care team has a culture of cost containment (keeping costs to a minimum, without reducing quality).

## How to generate income

It is important that the actual cost of the service (e.g. cataract surgery, outpatient consultation or reading spectacles) is calculated. Include all the ongoing costs in this calculation: salaries, consumables, water, electricity, rent, maintenance and depreciation costs. Once the full cost of the service is known, then you can determine how much income is needed in order to continue to provide the service.

**ABOUT THIS ISSUE**

**Figure:**
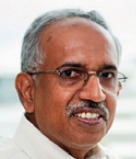
Thulasiraj Ravilla

Executive Director: Lions Aravind Institute of Community Ophthalmology, Aravind Eye Care System, Madurai, India. **thulsi@aravind.org**

Financial sustainability is probably the most important aspect of organisational sustainability, mainly because of its immediate impact: when funds are not available, activities can come to a grinding halt quite quickly.

Many of us have also learnt the hard way that external financial dependence can undermine the growth of an organisation, and even threaten its very existence – particularly when it is dependent on funding for day-to-day functioning.

In the face of ageing populations and existing inadequacies of care (including a shortage of health promotion and disease prevention services), eye care services and organisations not only have to sustain themselves in the years to come, but also to expand significantly to meet growing needs. To address this urgent topic, this issue shares case studies and suggests ways to reduce costs (including by enhancing productivity), generate income, and efficiently manage supply chains to ensure uninterrupted services; all while keeping the focus firmly on quality.

‘Full cost recovery’ is an often-heard financial term; it involves a costing exercise that allows you to work out what your services cost, in full.^1^

Income can be generated from a variety of sources:

1. Within the hospital.
2. Within the community.
3. At the national level.
4. Internationally.

The closer the source of income is to you as the service provider, the more self-reliance and self-determination you will have. If the income source is further away (for example, if you are being funded nationally or internationally), you can increase your self-reliance and self-determination by developing additional sources of income, closer to ‘home’, i.e. within the hospital or the community. Some ideas are discussed below.

### 1 Within the hospital

**The patient receiving the service.** The patient may pay the full cost of the service, be it a consultation, treatment or operation. If the patient cannot pay then a subsidy from another source is required.

**Other eye patients.** Some hospitals charge according to their patients’ income or ability to pay. Charging some patients more than the actual cost of the service allows the hospital to offer the same service at a below-cost price to patients who would otherwise be unable to afford it. This is known as a ‘tiered’ service: the same service is given to different people at a different price. With this model, because some are charged less and some are charged more, it may appear that income will not increase. However, by making treatment more affordable, the hospital will attract more patients and therefore the overall income is likely to increase, due to economies of scale.

Another approach is for additional non-clinical services to be offered at an additional cost. For example, a person with plenty of money may be given the option to pay an additional fee to have a private consultation at a time to suit them, and be hospitalised in a private single room with air conditioning, an en suite bathroom and internet access. The price paid for these non-clinical services can be used to subsidise consultations, treatments and operations for patients who cannot pay. The model is similar to air travel – one may travel first class, business class or economy class, depending on the available funds! However, it is important that the clinical service is of the same high quality for everyone; in air travel the pilot is the same, regardless of the class of travel.

**Figure:**
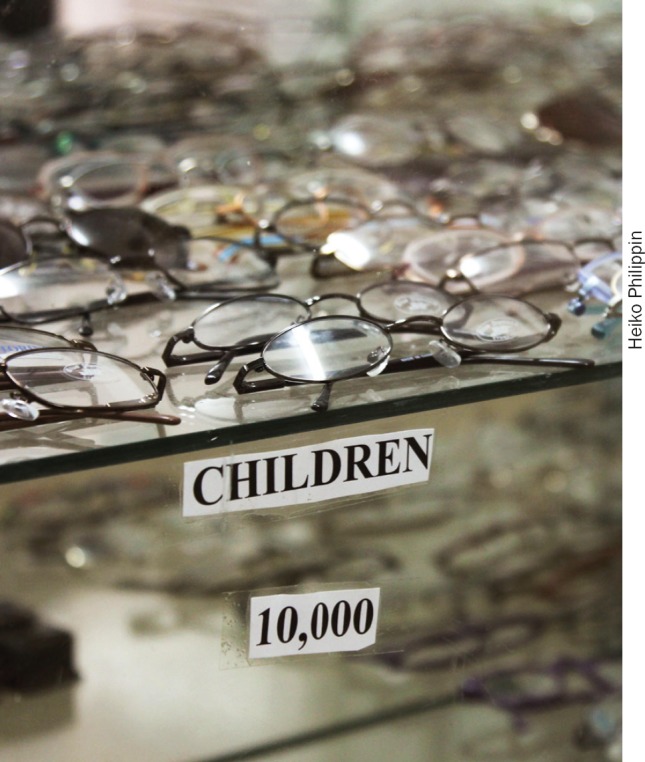
Sale of spectacles can support income generation.

**Sales of spectacles or eye medicines.** Ready-made spectacles can be purchased or made on site and sold at a profit. Similarly, eye medicines can be bought in bulk and dispensed to patients, also at a profit.

**Every little bit helps**

If one dispenses 40 pairs of spectacles a day for 250 working days per year at a profit of £2 per pair, the income generated is £20,000, which will subsidise 400 cataract operations at £50 per operation.

**Creating small business to generate income.** The hospital may generate income from small businesses (e.g. a tea or coffee shop, a restaurant, or by providing accommodation for the relatives of patients).

It is essential that any activities that support income generation are well managed in order to ensure that the quality of services remain high.

### 2 Within the community

**Local philanthropy.** Individuals or local service groups within the community may agree to subsidise outreach clinics, eye surgery for poor patients, or even the purchase of equipment or buildings.

**Corporate sponsorship.** Similarly, local businesses may agree to sponsor the eye services as part of their commitment to corporate social responsibility, particularly if the service can promote their name or logo (their ‘brand’).

Sometimes, local service organisations, businesses or individuals may want to help directly by providing volunteers. This can have its own challenges, but one should try and use volunteers as best as possible, while ensuring that the quality of services remain high. Volunteers can help translate or help illiterate patients to fill out forms. They can also run a refreshment stall, with the profit going to the eye clinic.

**Donations.** Some pharmaceutical companies, or other businesses involved in eye care, may provide appropriate donations of essential medicines or equipment. These can be used to lower the cost of the service to low-income patients. Previous issues of the *Community Eye Health Journal* have contained useful advice for making the most of such donations.^2^, ^3^

### 3 At the national level

**Government.** Ministries of health may provide all the running costs, or they may only provide the salaries of some staff, or a ‘bed or service’ grant. Governments are responsible for health service provision to the population and wherever possible they should be requested and encouraged to provide the funds for eye care services.

**National NGOs, foundations or corporations.** There may be a national NGO with a mandate to improve eye care which can fund some of the services. Alternatively, a foundation or corporation may be willing to sponsor the service, although this is more likely to be possible for development costs, rather than service provision (running) costs.

**Insurance schemes.** Some countries have insurance schemes for all or part of the population, which can be used to subsidise or pay for the cost of the service.

### 4 Internationally

**Bilateral or multilateral development aid.** Government-to-government aid may fund eye services. Examples are onchocerciasis and trachoma control activities funded through the World Bank and the UK Department for International Development (DfID). This is usually administered by the ministry of health.

**International NGOs.** There are many international NGOs (INGOs) which provide support for eye care services. As far as possible it is best to use this support for development costs such as staff training, or one-off equipment costs. It may also be useful for initial start-up costs, until a programme is able to cover its monthly or annual expenditure by generating its own income from patients, the community and national sources. Long-term reliance on INGOs for running costs does not promote financial sustainability and independence.

## How to keep quality high

While taking the above steps to reduce costs and increase income, it is essential that the quality of service and patient satisfaction are maintained at the highest levels. Ongoing monitoring of treatment outcomes and patient satisfaction makes it possible to deal with any problems as they arise and to ensure that quality remains high. Previous issues of the *Community Eye Health Journal* contain useful advice for monitoring clinical outcomes and patient satisfaction.^4^, ^5^

## Top tips to achieve financial sustainability

- Use what you have well, before looking for more resources.
- Aim to contain costs by ensuring only essential, generic consumables are bought – in bulk.
- Employ only essential staff who are well trained and have a clear job description.
- Promote a positive work culture through objective setting, regular feedback on performance and non-monetary incentives.
- Develop sources of income closer to you, the direct service provider – this will increase the degree of self-determination available to your programme.
- Generate income by charging for clinical and non-clinical services, to the extent that you are free to do so.
- Look to the local community and insurance schemes (if available) for additional sources of income.
- Use external support to pay for project start-up costs or to meet development costs such as staff training or the purchasing of equipment and instruments.

The suggestions in this article may be easier to apply in the non-government sector than in the government sector; however, the principles of cost containment and income generation are true for all sectors. How one applies them requires adaptation and perhaps a little innovation!
